# Impact of novel NS5A resistance-associated substitutions of hepatitis C virus detected in treatment-experienced patients

**DOI:** 10.1038/s41598-019-42114-z

**Published:** 2019-04-05

**Authors:** Sayuri Nitta, Yasuhiro Asahina, Takanobu Kato, Jun Tsuchiya, Emi Inoue-Shinomiya, Ayako Sato, Tomoyuki Tsunoda, Masato Miyoshi, Fukiko Kawai-Kitahata, Miyako Murakawa, Yasuhiro Itsui, Mina Nakagawa, Seishin Azuma, Sei Kakinuma, Hayato Hikita, Tetsuo Takehara, Mamoru Watanabe

**Affiliations:** 1 Department of Gastroenterology and Hepatology, Tokyo Medical and Dental University (TMDU), Tokyo, Japan; 2 Department of Liver Disease Control, Tokyo Medical and Dental University (TMDU), Tokyo, Japan; 3 Department of Virology II, National Institute of Infectious Diseases, Tokyo, Japan; 4 Department of Gastroenterology and Hepatology, Graduate School of Medicine, Osaka University, Osaka, Japan

## Abstract

Resistance-associated substitutions (RASs) of hepatitis C virus (HCV) in the NS5A region impair the efficacy of NS5A inhibitors. In this study, we evaluated the characteristics of the novel RASs observed in treatment-failure patients, A92K and a deletion at P32 (P32del), and the susceptibility of viruses with these RASs to various anti-HCV reagents by using JFH-1 based recombinant HCV with NS5A from a genotype 1b Con1 strain (JFH1/5ACon1). We introduced A92K or P32del solely or in combination with Q24K, L28M, R30Q or L31F into the NS5A of JFH1/5ACon1. Viruses harboring R30Q/A92K showed high extracellular core antigens and infectivity titers, whereas the other viruses with RASs showed low replication levels and infectivity titers. All the viruses with A92K or P32del were markedly resistant to ledipasvir, velpatasvir and elbasvir. Interestingly, viruses with R30Q/A92K were more susceptible to grazoprevir than viruses without RAS. All the viruses had a similar susceptibility to ribavirin and sofosbuvir. In conclusion, combination RASs R30Q/A92K enhanced virus production whereas other RASs impaired virus replication. Both A92K and P32del conferred severe resistance even to second generation NS5A inhibitors. However, these viruses were susceptible to grazoprevir, ribavirin and sofosbuvir. Thus, combination regimens with these reagents may eradicate viruses harboring A92K or P32del.

## Introduction

Hepatitis C virus (HCV) infection is a global health problem. The number of people infected with HCV is approximately 80 million worldwide^1,2^. Of note, HCV infection leads to chronic hepatitis, cirrhosis, end-stage liver dysfunction and hepatocellular carcinoma. The global eradication of this virus is the ultimate goal to solve HCV-associated health problems. For this purpose, many anti-viral reagents have been investigated, and novel reagents designated direct-acting antiviral agents (DAAs) that inhibit viral proteins directly have been established^3^. The use of DAAs has markedly improved the sustained virological response (SVR) rate of anti-HCV treatments^1,4^. Among currently available DAAs, NS5A inhibitors have potent anti-HCV effects and are described as key reagents that are included in many recommended DAA regimens in the treatment guidelines for hepatitis C^5–7^. However, the potency of NS5A inhibitors has been impaired by the emergence of specific amino acid substitutions designated resistance-associated substitutions (RASs)^8–10^. RASs of L31F/M/V/I and Y93H in the NS5A region are frequently observed in both DAA naïve and experienced patients infected with HCV genotype 1b strains^9,11–13^. These RASs were reported to be associated with virologic failure of the DAA therapies including first generation NS5A inhibitors, such as daclatasvir (DCV), ledipasvir (LDV) and ombitasvir^9,14^. To achieve a high SVR rate, even in patients with these RASs, second generation NS5A inhibitors were developed and their superior efficacy was reported^15–17^. However, in recent studies, novel RASs in the NS5A region, A92K or a deletion at P32 (P32del), were identified^18–20^. These novel RASs were not detected in patients before treatment but emerged after DAA treatment alone or in combination. These RASs are considered resistant to DAAs, but the resistance levels, especially in combination with other RASs, have not been investigated. Therefore, the evaluation of these RASs in terms of resistance to DAAs and the identification of effective compounds for these RASs are urgent issues.

HCV subgenomic replicon system have been used in many studies to investigate RASs. However, this system is not able to produce infectious virus and is not suitable for evaluating the HCV whole life cycle. Whereas, HCV cell culture system enables to assess the virus whole life cycle. Previously, we used a recombinant virus of JFH-1 to assess the effects of the RASs, L31M/V/I and Y93H, on the replication and infectious virus production of HCV and to identify effective DAAs to these RASs^21^. The used recombinant virus has NS5A from the genotype 1b strain and enabled the assessment for the effects of RASs on the HCV life cycle and susceptibility to anti-HCV reagents against genotype 1b strains. The current study used this system to evaluate the effects of the novel RASs A92K and P32del on the HCV life cycle and susceptibility to various anti-HCV reagents.

## Results

### Effects of A92K Substitution on the HCV Life Cycle

**Figure 1:**
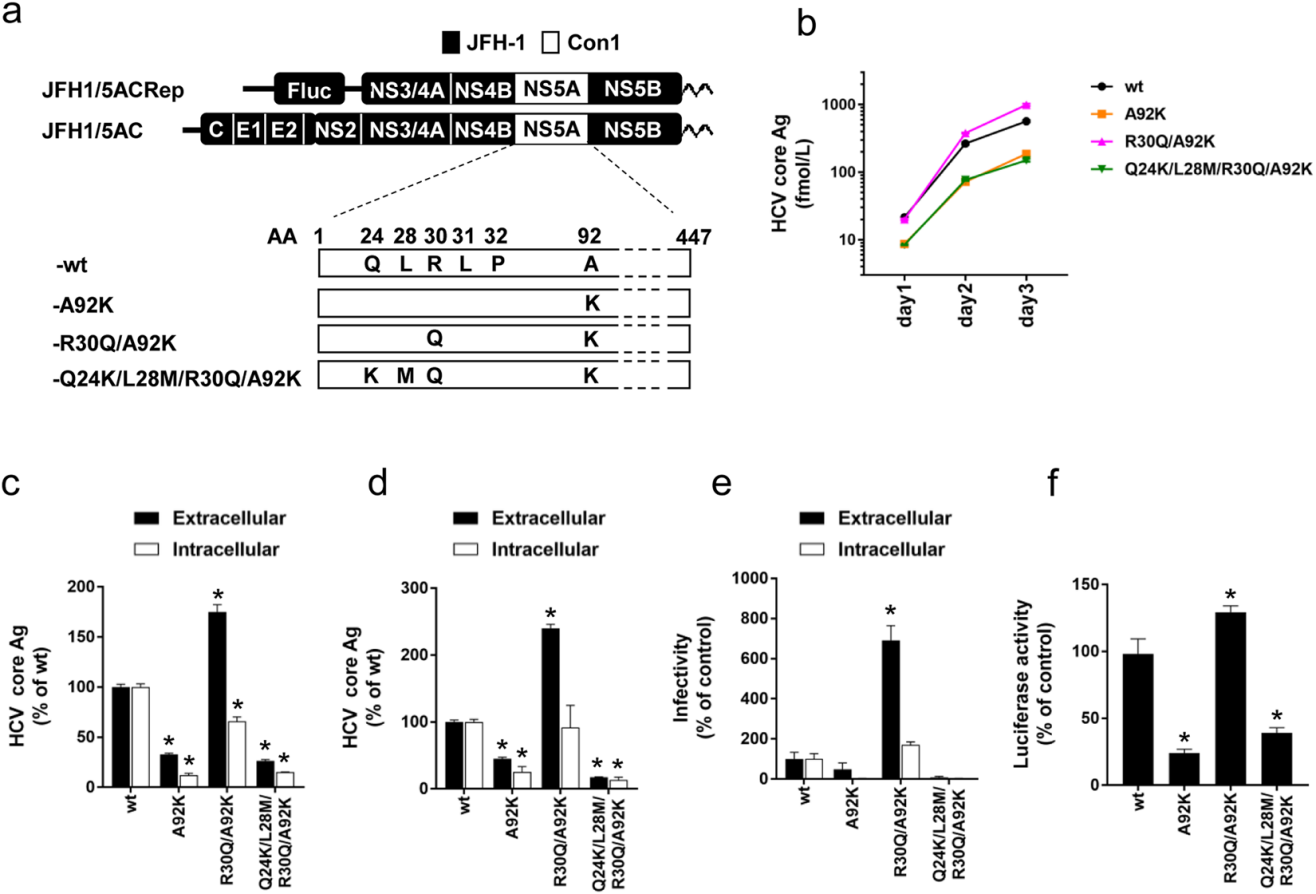
Characteristics of the recombinant virus JFH1/5AC with A92K. (**a**) Schematic representation of the recombinant virus (JFH1/5AC), subgenomic replicon (JFH1/5ACRep) and their derivatives with NS5A resistance-associated substitutions. (**b**) Time course of extracellular core Ag levels of HCV RNA-transfected Huh7.5.1 cells. (**c**,**d**) *In vitro* transcribed HCV RNAs were electroporated into Huh 7.5.1 (**c**) or Huh7-25 (**d**) cells. On day 3 post-transfection, extra- and intracellular core Ag levels were quantified. (**e**) Extra- and intracellular infectivity titers of HCV RNA-transfected Huh7-25 cells. (**f**) Luciferase activity of HCV-subgenomic replicon RNA-transfected Huh-7.5.1 cells on day 3 post-electroporation. Replication levels were calculated as fold increases at 4 hours. (**c**–**f**) Data are expressed as the percentages of JFH1/5AC-wt. *p < 0.05.

To assess the effect of A92K on HCV propagation, we used the JFH-1 based recombinant virus with the NS5A of genotype 1b strain (JFH1/5AC-wt)^21,22^. A92K was introduced in this virus alone (JFH1/5AC-A92K) or in combination with R30Q (JFH1/5AC-R30Q/A92K) or Q24K, L28M and R30Q (JFH1/5AC-Q24K/L28M/R30Q/A92K) (Fig. 1a). We transfected the full-length RNAs of these viruses into Huh7.5.1 cells and measured the HCV core antigen (Ag) levels on days 1, 2 and 3 post-transfection. Time-dependent increases of extracellular HCV core Ag levels were observed in these cells, but the levels were different between recombinant viruses (Fig. 1b). The extracellular core Ag levels of JFH1/5AC-wt and -R30Q/A92K were higher than that of the other recombinant viruses. In JFH1/5AC-R30Q/A92K transfected cells, the extracellular core Ag level was higher, but the intracellular core Ag level was slightly lower than that of JFH1/5AC-wt at day 3 post-transfection (Fig. 1c). In addition, both the extra- and intracellular HCV core Ag levels of JFH1/5AC-A92K and -Q24K/L28M/R30Q/A92K were substantially lower than that of JFH1/5AC-wt. To explore the steps in the HCV life cycle affected by the RASs, we performed a single cycle virus production assay with Huh7-25 cells. Huh7-25 cell line supports HCV replication and virus production after the transfection of full-length HCV RNA, but it is not re-infected with progeny viruses because it lacks the expression of CD81 on the cell surface^23^. Therefore, it allows to assess which steps were affected in a single cycle of virus production. The results of this experiment were similar with that using Huh-7.5.1 cells; the HCV core Ag levels of JFH1/5AC-R30Q/A92K were approximately 2.5-fold higher in culture medium and were comparable in cell lysates compared with JFH1/5AC-wt (Fig. 1d). Both the extra- and intracellular HCV core Ag levels of other viruses were less than 0.5-fold that of JFH1/5AC-wt.

To assess the infectivity of the viruses with these RASs, we next evaluated the extra- and intracellular infectivity titers of these recombinant viruses. Extracellular infectivity titers of JFH1/5AC-R30Q/A92K transfected cells were more than 7-fold higher than that of JFH1/5AC-wt, whereas few HCV infected cells were observed in the infection of culture medium and cell lysates from JFH1/5AC-A92K and -Q24K/L28M/R30Q/A92K transfected cells (Fig. 1e and Supplementary Fig. S1). These results suggest that substitutions of R30Q/A92K enhanced the production levels of infectious viruses, and that substitutions of A92K and Q24K/L28M/R30Q/A92K attenuated the virus replication.

To confirm the attenuated replication efficiencies of JFH1/5AC-A92K and -Q24K/L28M/R30Q/A92K, we used the JFH-1 based recombinant subgenomic replicon system (JFH1/5ACRep). Similar to the full-length recombinant viruses, we introduced the RASs of A92K, R30Q/A92K and Q24K/L28M/R30Q/A92K and generated JFH1/5ACRep-A92K, -R30Q/A92K and -Q24K/L28M/R30Q/A92K, respectively (Fig. 1a). *In vitro* transcribed replicon RNAs were electroporated into Huh7.5.1 cells and luciferase activities were measured on day 3 after electroporation (Fig. 1f). The luciferase activities of JFH1/5ACRep-A92K and -Q24K/L28M/R30Q/A92K were significantly lower than those of JFH1/5ACRep-wt and those of JFH1/5ACRep-R30Q/A92K were slightly higher than -wt. These results were consistent with the results of the intracellular HCV core Ag levels of full-length HCV transfected Huh7-25 cells. These results indicate that A92K reduced virus replication but that R30Q compensated for it mainly by enhancing infectious virus production and partially by enhancing replication.

### Effects of P32del Substitution on the HCV Life Cycle

**Figure 2:**
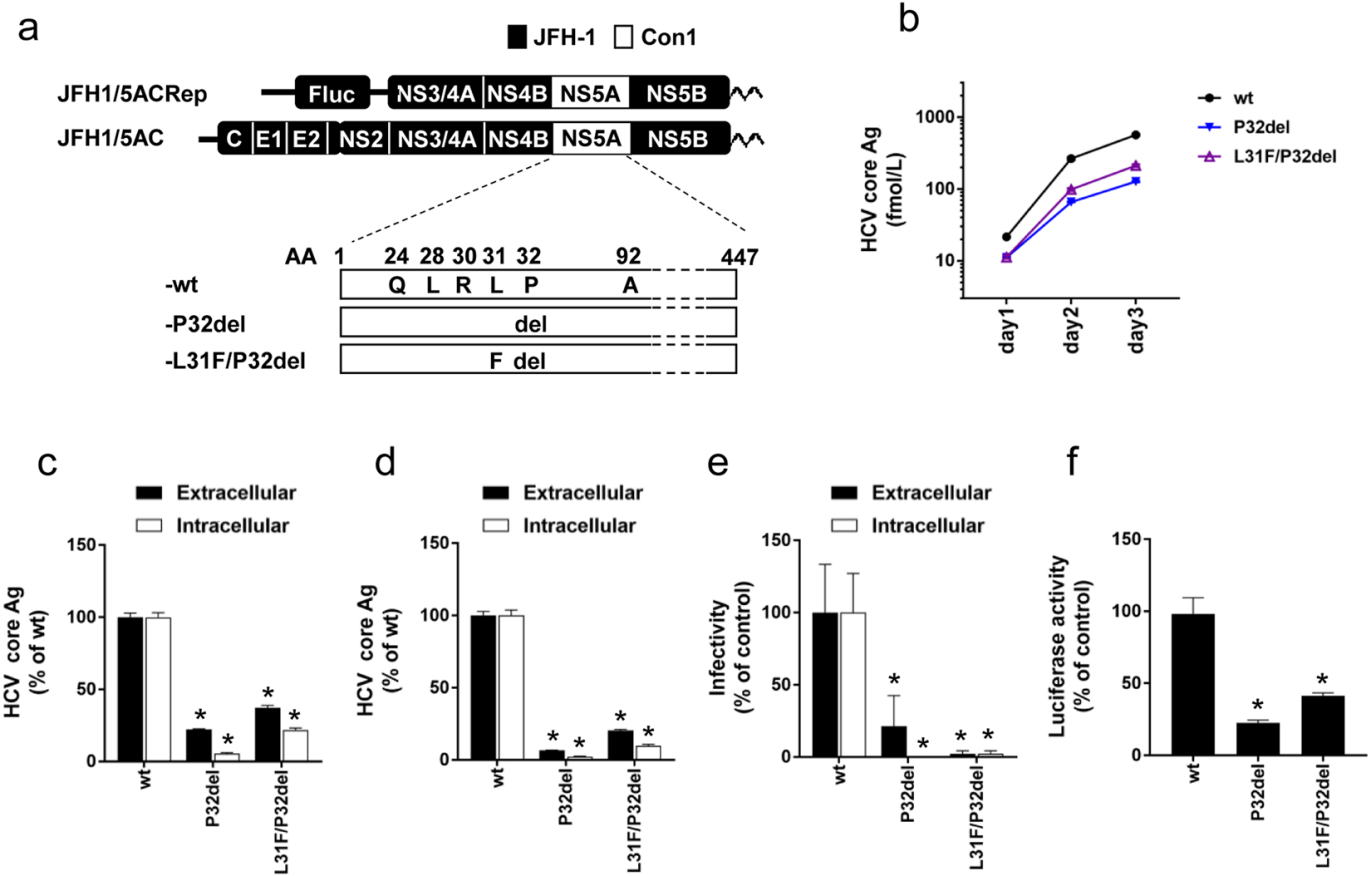
Characteristics of the recombinant virus JFH1/5AC with P32del. (**a**) Schematic representation of the recombinant virus (JFH1/5AC), subgenomic replicon (JFH1/5ACRep) and their derivatives with NS5A resistance-associated substitutions. (**b**) Time course of extracellular core Ag levels of HCV RNA-transfected Huh7.5.1 cells. (**c**,**d**) *In vitro* transcribed HCV RNAs were electroporated into Huh7.5.1 (**c**) or Huh7–25 (**d**) cells. On day 3 post-transfection, extra- and intracellular core Ag levels were quantified. (**e**) Extra- and intracellular infectivity titers of HCV RNA-transfected Huh7-25 cells. (**f**) Luciferase activity of HCV-subgenomic replicon RNA-transfected Huh-7.5.1 cells on day 3 post-electroporation. Replication levels were calculated as fold increases at 4 hours. (**c**–**f**) Data are expressed as the percentages of JFH1/5AC-wt. *p < 0.05.

To evaluate the effect of P32del on the virus life cycle, we used JFH1/5AC-wt by introducing P32del alone (JFH1/5AC-P32del) or in combination with L31F (JFH1/5AC-L31F/P32del) (Fig. 2a). When Huh7.5.1 cells were transfected with the full-length RNAs of these viruses, the extracellular HCV core Ag levels of JFH1/5AC-P32del and -L31F/P32del increased in a time-dependent manner although the levels were lower than that of JFH1/5AC-wt (Fig. 2b). At day 3 post-transfection, both the extra- and intracellular core Ag levels of JFH1/5AC-P32del and -L31F/P32del were substantially lower than that of JFH1/5AC-wt (Fig. 2c). The core Ag levels of JFH1/5AC-L31F/P32del were slightly higher than that of JFH1/5AC-P32del. To evaluate which steps in the HCV life cycle were affected by these RASs, we performed the single cycle virus production assay (Fig. 2d). In this experiment, the results were similar to that with Huh-7.5.1 cells. Both the extra- and intracellular HCV core Ag levels of JFH1/5AC-P32del and -L31F/P32del were less than approximately 0.2-fold that of JFH1/5AC-wt and the HCV core Ag levels of JFH1/5AC-L31F/P32del were more than 3-fold higher than JFH1/5AC-P32del. The results suggest the attenuated replication efficiencies of these viruses and complementary effect of L31F on the reduced replication of JFH1/5AC-P32del. We next assessed the extra- and intracellular infectivity titers of these recombinant viruses. Few HCV infected cells were detected by infection with culture medium and cell lysates from JFH1/5AC-P32del and -L31F/P32del transfected cells (Fig. 2e and Supplementary Fig. S1). To confirm the attenuated replication efficiencies of JFH1/5AC-P32del and -L31F/P32del, we generated the subgenomic replicons corresponding to these viruses (JFH1/5ACRep-P32del and -L31F/P32del) and performed the luciferase assay (Fig. 2a and 2f). The luciferase activities of JFH1/5ACRep-P32del and -L31F/P32del were significantly lower than that of JFH1/5ACRep-wt. These results were consistent with the data of the single cycle virus production assay.

### Susceptibility of JFH1/5AC with RASs to NS5A inhibitors

**Figure 3:**
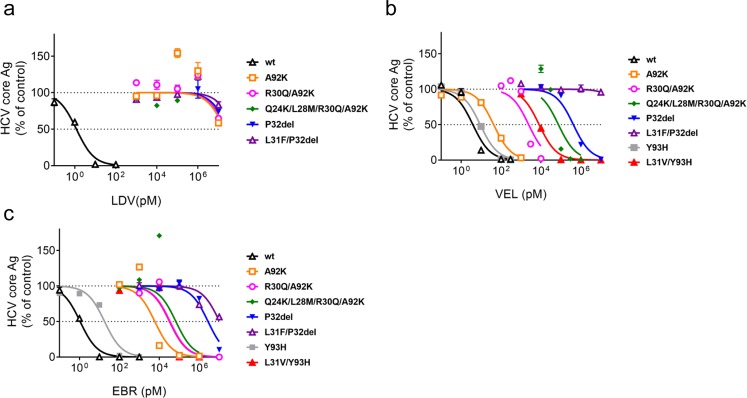
Susceptibility of JFH1/5AC and its derivatives with NS5A RAS to NS5A inhibitors. *In vitro* transcribed HCV RNAs of JFH1/5AC-wt and indicated derivatives were electroporated into Huh-7.5.1 cells. Four hours after transfection, the cells were treated with LDV (**a**) VEL (**b**) or EBR (**c**). After incubation for 72 hours, the supernatants were harvested and extracellular HCV core Ag levels were quantified. The data are expressed as the percentages of the DMSO-treated control.

To verify the conferred resistance of A92K, P32del and associated RASs to NS5A inhibitors, we assessed the susceptibility of JFH1/5AC-A92K, -R30Q/A92K, -Q24K/L28M/R30Q/A92K, -P32del and -L31F/P32del to LDV, VEL or EBR. The resistance levels of these recombinant viruses were compared with JFH1/5AC-wt, Y93H introduced JFH1/5AC (JFH1/5AC-Y93H) or L31V/Y93H introduced (JFH1/5AC-L31V/Y93H). We treated the HCV RNA-transfected cells with LDV, EBR or VEL for 72 hours and measured the extracellular HCV core Ag levels to determine the EC50 values. Treatment with NS5A inhibitors inhibited the HCV core Ag level of JFH1/5AC-wt at a low concentration. The EC50 values of JFH1/5AC-wt to these NS5A inhibitors were comparable and were 1.1–3.9 pM (Fig. 3 and Table 1). However, all recombinant viruses with A92K or P32del exhibited marked resistance to LDV, and the EC50 values were higher than 20 μM (Fig. 3a and Table 1). For the treatment with VEL or EBR, JFH1/5AC-R30Q/A92K and -Q24K/L28M/R30Q/A92K showed marked resistance comparable to or higher than for JFH1/5AC-L31V/Y93H. The EC50 values of JFH1/5AC-R30Q/A92K, -Q24K/L28M/R30Q/A92K and - L31V/Y93H for VEL were 2.4 × 10^3^, 7.5 × 10^4^, and 8.1 × 10^3^ pM, respectively, and for EBR they were 3.4 × 10^4^, 6.8 × 10^4^ and 3.2 × 10^4^ pM, respectively (Fig. 3b,c and Table 1). Interestingly, JFH1/5AC-A92K exhibited a greater resistance to LDV and EBR compared with VEL, and JFH1/5AC-P32del had a marked resistance to all NS5A inhibitors. Moreover, JFH1/5AC-L31F/P32del had a greater resistance to VEL and EBR compared with JFH1/5AC-P32del, and the EC50 values were 6.3 × 10^7^ pM for LDV, 2.3 × 10^8^ pM for VEL and 1.0 × 10^7^ pM for EBR (Fig. 3a–c and Table 1).

**Table 1:** EC50 values of JFH1/5ACon1 and its derivatives with NS5A RAS to NS5A inhibitors.

Strain (JFH1/5ACon1-)	LDV		VEL		EBR	
	EC50 (pM) (95%CI)	Fold Resistance	EC50 (pM) (95% CI)	Fold Resistance	EC50 (pM) (95% CI)	Fold Resistance
wt	1.2 (0.8–1.7)	1.0	3.9 (2.3–6.6)	1.0	1.1 (0.8–1.4)	1.0
A92K	2.0 × 10^7^ (3.6 × 10^6^–1.1 × 10^8^)	1.7 × 10^7^	42.6 (30.8–58.8)	13.7	6.0 × 10^3^ (2.8 × 10^3^–1.3 × 10^4^)	8.6 × 10^3^
R30Q/A92K	2.4 × 10^7^ (8.8 × 10^6^–3 × 10^7^)	2.0 × 10^7^	2.4 × 10^3^ (1.2 × 10^3^–4.9 × 10^3^)	6.2 × 10^2^	3.4 × 10^4^ (1.5 × 10^4^–8.0 × 10^4^)	3.1 × 10^4^
Q24K/L28M/R30Q/A92K	2.0 × 10^7^ (7.6 × 10^6^–5.4 × 10^7^)	1.7 × 10^7^	7.5 × 10^4^ (3.0 × 10^4^–1.9 × 10^5^)	1.9 × 10^4^	6.8 × 10^4^ (9.6 × 10^3^–4.8 × 10^5^)	6.2 × 10^4^
P32del	3.1 × 10^7^ (1.5 × 10^7^–6.1 × 10^7^)	2.6 × 10^7^	4.2 × 10^5^ (3.0 × 10^4^–5.9 × 10^5^)	1.1 × 10^5^	2.8 × 10^6^ (1.8 × 10^6^–4.3 × 10^6^)	2.5 × 10^6^
L31F/P32del	6.3 × 10^7^ (1.6 × 10^7^–2.5 × 10^8^)	5.3 × 10^7^	2.3 × 10^8^ (3.6 × 10^7^–1.5 × 10^9^)	5.9 × 10^6^	1.0 × 10^7^ (6.3 × 10^6^–1.7 × 10^7^)	9.1 × 10^6^
Y93H	^†^3.7 × 10^3^ (2.7 × 10^3^–4.8 × 10^3^)	^†^5.8 × 10^3^	9.0 (7.1–11.5)	2.3	18.1 (11.7–28.0)	16.5
L31V/Y93H	^†^3.5 × 10^5^ (2.4 × 10^5^–4.6 × 10^5^)	^†^5.4 × 10^5^	8.1 × 10^3^ (6.6 × 10^3^–9.8 × 10^3^)	2.1 × 10^3^	3.2 × 10^4^ (1.4 × 10^4^–7.4 × 10^4^)	2.9 × 10^4^

LDV; ledipasvir, VEL; velpatasvir, EBR; elbasvir, EC50; effective concentrations required to inhibit 50% of extracellular core protein level, CI; confidence interval. ^†^Data from^21^.

These results suggest that A92K substitution was responsible for the resistance of JFH1/5AC-R30Q/A92K and -Q24K/L28M/R30Q/A92K to NS5A inhibitors and that P32del conferred a marked resistance to NS5A inhibitors.

### Susceptibility of JFH1/5AC with RASs to the other anti-HCV reagents

**Figure 4:**
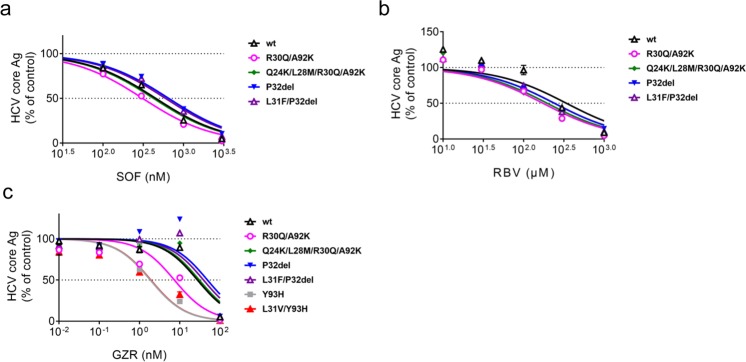
Susceptibility of JFH1/5AC and its derivatives with NS5A RAS to SOF, RBV and GZR. *In vitro* transcribed HCV RNAs of JFH1/5AC-wt and indicated derivatives were electroporated into Huh-7.5.1 cells. Four hours after transfection, the cells were treated with SOF (**a**) RBV (**b**) or GZR (**c**). After incubation for 72 hours, the supernatants were harvested and extracellular HCV core Ag levels were quantified. Data are expressed as the percentages of the DMSO-treated control.

To evaluate the susceptibilities of recombinant viruses with RASs to the other anti-HCV reagents, we measured the extracellular HCV core Ag levels after treatment with SOF, RBV or GZR. The core Ag levels of viruses with RASs were reduced by treatment with SOF, RBV or GZR in a concentration-dependent manner (Fig. 4a–c). When treated with SOF or RBV, the dose-response curves of viruses with RASs were similar to that of JFH1/5AC-wt (Fig. 4a,b), and the EC50 values of these strains were comparable (Table 2). These data suggest that the inhibitory effect of SOF and RBV on these strains with NS5A RASs were comparable to JFH1/5AC-wt.

**Table 2:** EC50 values of JFH1/5ACon1 and its derivatives with NS5A RAS to SOF, RBV and GZR.

Strain (JFH1/5ACon1-)	SOF		RBV		GZR	
	EC50 (nM) (95% CI)	Fold Resistance	EC50 (μM) (95% CI)	Fold Resistance	EC50 (nM) (95% CI)	Fold Resistance
wt	452.4 (345.4–592.5)	1.0	340.5 (169.7–683.2)	1.0	27.5 (15.0–50.5)	1.0
R30Q/A92K	424.4 (337.8–533.2)	0.9	197.1 (112.9–344.2)	0.6	7.4 (3.8–14.2)	0.3
Q24K/L28M/R30Q/A92K	655.3 (538.3–797.8)	1.4	241.1 (178.9–325.0)	0.7	30.6 (15.7–59.8)	1.1
P32del	598.3 (469.6–762.2)	1.3	242.3 (160.7–365.4)	0.7	48.8 (15.8–151.1)	1.8
L31F/P32del	452.4(345.4–592.5)	1.0	340.5 (169.7–683.2)	1.0	38.9 (16.9–89.9)	1.4
Y93H	—	—	—	—	1.9 (1.3–2.6)	0.1
L31V/Y93H	—	—	—	—	1.9 (1.0–3.5)	0.1

SOF; sofosbuvir, RBV; ribavirin, GZR; grazoprevir, EC50; effective concentrations required to inhibit 50% of extracellular core protein level, CI; confidence interval.

We previously demonstrated that viruses with Y93H were more susceptible to the protease inhibitors, simeprevir and asunaprevir than viruses without RASs^21^. Therefore, we evaluated the susceptibility of RAS introduced viruses including JFH1/5AC-Y93H and -L31V/Y93H to GZR, a novel protease inhibitor. As expected, treatment with GZR reduced the extracellular HCV core Ag levels of JFH1/5AC-Y93H and -L31V/Y93H more efficiently than that of JFH1/5AC-wt (Fig. 4c). Interestingly, the reduction of HCV core Ag levels of JFH1/5AC-R30Q/A92K was also more efficient in comparison with JFH1/5AC-wt and the dose-response curve of this strain shifted to the left suggesting a higher susceptibility of this virus to GZR. The EC50 value of JFH1/5AC-R30Q/A92K was 7.4 nM and was significantly lower than that of JFH1/5AC-wt; however, its susceptibility level was not pronounced compared with the levels of JFH1/5AC-Y93H and -L31V/Y93H. The EC50 values of these viruses to GZR were both 1.9 nM and were 0.1-fold that of JFH1/5AC-wt (Table 2). The EC50 values of the other viruses with RASs were similar or slightly, but not significantly, higher than that of JFH1/5AC-wt.

## Discussion

NS5A inhibitors play a pivotal role in the treatment of chronic hepatitis C. NS5A RASs reduced the treatment efficacy and had a great impact on the selection of therapeutic regimens when the 1st generation NS5A inhibitors mainly used^9,14,24^. Although the recently developed DAA combination therapies including 2nd generation NS5A inhibitors have been reported to eradicate the viruses with NS5A RASs and to have high efficacies, specific or multiple RASs is still associated with the treatment failure especially for cirrhosis patients^25^. The most described NS5A RASs of L31F/M/V/I and Y93H in genotype 1b are often detected in treatment naïve patients at ~6% and ~10%, respectively^9,11–13^. On the other hand, the prevalence of these RASs extremely increases in DAA-experienced patients up to 40% and 60%, respectively^18,26^. RAS at amino acid (aa) 24, aa 28 and aa 30 in the NS5A region detected in DAA-experienced patients also increase ~4-folds compared with treatment-naïve patients. In addition, recent studies have reported the emergence of novel RASs: A92K and P32del. These RASs were not detected in treatment naïve patients but emerged at the time of the treatment failure of DAA therapy at ~5% treatment with ASV and DCV and also LDV and SOF^19,20,27–30^. Of note, virus harboring P32del have been reported to difficult to eliminate by retreatment with LDV and SOF or glecaprevir and pibrentasvir^31,32^. Furthermore, in many cases, these NS5A RASs emerge in combination with multiple RASs in DAA-experienced patients^18^. Based on these backgrounds, we reasoned that the information of NS5A RASs should not be ignored when considering the treatment strategy. Thus, the present study evaluated the effects of these novel RASs on the HCV life cycle by exploiting the HCV cell culture system with JFH1 based NS5A chimeric viruses and assessed the susceptibility to various anti-HCV reagents.

The assessment of effects on the viral life cycle indicated that chimeric viruses harboring A92K and P32del had attenuated fitness. Viruses with these RASs had a lower replication efficiency compared with JFH1/5AC-wt. This finding may explain why these RASs are rarely detected in treatment naïve patients at baseline^9,12,18,29,33^. In the chimeric virus harboring A92K, the addition of R30Q compensated for the virus propagation by enhancing infectious virus production and may partially by enhancing virus replication (Fig. 1). A similar effect was observed by introducing mutations at the same positions in the genotype 2a JFH-1 strain^34^. The mutation of K30E/Q compensated for the impaired replication efficiency induced by the C92R mutation in the JFH-1 strain. Therefore, amino acids at these positions appear to coordinate with each other, and this effect is maintained beyond genotypes. The enhancement of infectious virus production by R30Q resembled the effect of Y93H reported in our previous study^21^. Therefore, similar to HCV with Y93H, HCV with R30Q/A92K may persist for a long period after treatment failure^21,35,36^. Notably, the observed compensation by R30Q disappeared by the addition of the Q24K/L28M mutations. JFH1/5AC-Q24K/L28M/R30Q/A92K exhibited lower replication and virus production efficiencies.

In the chimeric virus with P32del, the addition of L31F conferred a minor effect on the HCV life cycle. The replication efficiency of JFH1/5AC-L31F/P32del was slightly higher than that of -P32del and the production of infectious viruses were similar (Fig. 2). From these observations, we hypothesized that a virus with P32del will decrease and disappear soon after treatment failure. However, a recent report indicated that a virus with P32del persisted for at least one year after treatment similar to a virus with Y93H^20,37^. Thus, some unknown mutations might compensate for the attenuated viral fitness induced by P32del and enable the virus to persist.

Regarding the drug susceptibility analysis, we evaluated the resistance of these RASs introduced viruses to NS5A inhibitors (LDV, EBR, and VEL) and other anti-HCV reagents (SOF, RBV, and GZR). JFH1/5AC-wt was susceptible to the NS5A inhibitors with EC50 values ranging from 1.1 to 3.9 pM (Fig. 3 and Table 1). The observed values were consistent with previous reports^38–41^. However, viruses harboring the novel RASs of A92K or P32del exhibited high resistance to the conventional NS5A inhibitor, LDV. The EC50 values of these viruses to LDV were significantly higher than that of JFH1/5AC-Y93H or -L31V/Y93H reported in our previous paper^21^. The resistance levels of viruses with these RASs to the second generation NS5A inhibitors VEL and EBR were varied. JFH1/5AC-Y93H exhibited low-level resistance to EBR and no resistance to VEL in comparison with JFH1/5AC-wt. The addition of L31V to Y93H resulted in enhanced resistance and the EC50 value was increased to greater than 10^3^-fold that of JFH1/5AC-wt. The highest EC50 values for these reagents were observed in JFH1/5AC-L31F/P32del followed by -P32del. Resistance due to A92K alone was not potent but higher than that of Y93H, and the EC50 value of this virus was lower than that of JFH1/5AC-P32del. The combination of R30Q with A92K enhanced the resistance levels to NS5A inhibitors, but the EC50 value of this virus was still lower than that of JFH1/5AC-P32del and comparable to JFH1/5AC-L31V/Y93H. In clinical studies, the detection of a single A92K RAS is very rare in both treatment-naïve and treatment-experienced patients^19,33^. In treatment-experienced patients, A92K is often observed with R30Q or with Q24K/L28M/R30Q, whereas the detection of L31F/M/V/I or Y93H with A92K is very rare^19,33^. RASs of L28M or R30Q were often detected in approximately 10% of treatment naïve patients^9,29,33^. Thus, A92K is important for patients infected with viruses harboring L28M and/or R30Q but without L31F/M/V/I and/or Y93H viruses as an additional RAS that confers increased resistance to NS5A inhibitors. In contrast, a single mutation of P32del conferred marked resistance to NS5A inhibitors. As expected, none of the DAA-experienced patients with P32del could be rescued by combination therapy with the potent NS5A inhibitor pibrentasvir and the second generation protease inhibitor glecaprevir^31,42^.

Regarding anti-HCV reagents other than NS5A inhibitors, no difference was observed in susceptibility to SOF and RBV between viruses with NS5A RASs and without RASs (Fig. 4 and Table 2). Interestingly, we found that JFH1/5AC-R30Q/A92K was more susceptible to GZR than JFH1/5AC-wt as well as JFH1/5AC-Y93H and -L31V/Y93H. This suggests that GZR is a good treatment option for viruses harboring R30Q/A92K. Similarly, we previously demonstrated that viruses with Y93H were more susceptible to the NS3/4A protease inhibitors, simeprevir and asunaprevir^21^. Intriguingly, both viruses harboring R30Q/A92K and Y93H enhanced the production of infectious virus particles. There may be some association between high virus production and the strong effect of protease inhibitors. HCV NS3 protein was reported to be essential for viral assembly in cooperation with NS5A or other virus proteins^43–45^. Thus, protease inhibitors might have various levels of inhibitory effect on virus assembly processes through the NS3 protein that follows the recruitment of NS5A protein to produce virus particles. Because the recombinant viruses used in this study contained the NS3 of genotype 2a and NS5A of genotype 1b, it is unclear whether this result is applicable to genotype 1b viruses. Confirmation by clinical observation is required to verify these *in vitro* findings and future studies should determine the mechanisms involved in the anti-viral effects of protease inhibitors to these viruses. Taken together with our susceptibility analysis, treatment with SOF, RBV, or GZR may eradicate viruses harboring P32del or A92K.

In conclusion, our experiments using JFH1 based NS5A chimeric recombinant viruses clearly demonstrated that R30Q/A92K substitution in NS5A contributes to enhanced virus propagation and that this might cause persistent infection similar to the Y93H variant. To treat this virus, our data suggests a DAA regimen containing GZR will be a useful option. Furthermore, viruses with a deletion at P32 were strongly resistant to all NS5A inhibitors including VEL. However, the propagation levels of these variants were very low and they were susceptible to SOF, RBV, and GZR. Combination regimens containing these anti-HCV reagents other than NS5A inhibitors will be better options for treating patients infected with viruses harboring P32del.

## Materials and Methods

### Cell Culture

The HuH-7-derived cell lines Huh-7.5.1 (provided by Francis Chisari, Scripps Research Institute, La Jolla, CA, USA) and Huh7-25 were cultured in Dulbecco’s Modified Eagle’s Medium supplemented with 10% fetal bovine serum essential medium at 37 °C in a 5% CO_2_ as described previously^46^.

### Plasmid Construction

We used the JFH-1 based chimeric virus which NS5A was replaced with that from the Con1 strain (JFH1/5AC-wt) as described previously^21,22^. In this study, we focused on two novel RASs in NS5A, A92K and P32del. These RASs were not detected in DAA naïve patients but emerged at the time of the virologic failure of DAA treatment^18^. The RAS of A92K has been reported to emerge in combination with R30Q or with Q24K/L28M/R30Q^19,33^. Therefore, we made three variants of A92K by site-directed mutagenesis with appropriate primers as follows: JFH1/5AC-A92K, -R30Q/A92K and -Q24K/L28M/R30Q/A92K. The emergence of P32del was reported alone or in combination with L31F^19^. Therefore, we made two variants of P32del as follows: JFH1/5AC-P32del and - L31F/P32del. We also used the JFH-1 based subgenomic firefly luciferase reporter replicon with the NS5A from the Con1 strain (JFH1/5ACRep-wt), and constructed subgenomic reporter replicons with the RASs described above.

### RNA Transfection and Quantification of the HCV Core Antigen

RNA transfection and quantification of the HCV core Ag were performed as described previously^21^. Briefly, *in vitro* transcribed HCV RNAs were electroporated into cells. The transfected cells and the supernatants were harvested and the concentration of the HCV core Ags were quantified using a Lumipulse Ortho HCV Ag kit (Ortho Clinical Diagnostics, Tokyo, Japan) according to the manufacturer’s instructions. *In vitro* RNA synthesis and electroporation were performed as described previously^21,46^.

### Susceptibility Analysis for Antiviral Reagents

Susceptibility analysis for antiviral reagents were performed as described previously^21^. Briefly, *in vitro* transcribed HCV RNAs were electroporated into Huh-7.5.1 cells. Four hours after electroporation, transfected cells were treated with various concentrations of anti-HCV reagents. After incubation for 72 hours, the supernatants of the cells were harvested and the HCV core Ags were quantified. The anti-HCV reagents used were as follows: ribavirin (RBV) (Sigma-Aldrich, St. Louis, MO, USA), ledipasvir (LDV) (GS5885; AdooQ Bioscience, Irvine, CA, USA), elbasvir (EBR) (Chem Scene, Monmouth Junction, NJ, USA), velpatasvir (VEL) (Chem Scene), grazoprevir (GZR) (MK-5172; Chem Scene), and sofosbuvir (SOF) (PSI-7977; Medchemexpress LLC, NJ, USA). Dose-response curves were fitted using GraphPad Prism 7 software (GraphPad Software, Inc., CA, USA) and the values of the effective concentrations required to inhibit 50% (EC50) were determined with the results of the extracellular HCV core Ag quantification. The fitted model is as follows: Y = 100/(1 + 10^((X-LogIC50))^), where X is the concentration of the reagent and Y is the response expressed as the percentage of HCV core Ag related to the corresponding control.

### Titration of HCV Infectivity and Indirect Immunofluorescence Analysis

The HCV infectivity titers were measured by indirect immunostaining as described previously^21,46^. For the indirect immunofluorescence analysis, the cells were fixed with cold methanol and incubated with an anti-core antibody (clone C7–50; Abcam, Cambridge, UK) and subsequently with fluorescent labeled secondary antibody. The infectivity titer was calculated the focus-forming units per mL and expressed as relative to JFH1/5AC-wt.

### Subgenomic Replicon Analysis

*In vitro* transcribed HCV RNAs of subgenomic replicons were electroporated into Huh-7.5.1 cells. Four hours after transfection, the culture media were replaced with fresh media. After incubation for 3 days, cells were harvested and a luciferase assay was performed according to the manufacturer’s instructions (Bright-Glo™ Luciferase Assay System, Promega).

### Statistical Analysis

Statistical analysis was performed by two-way ANOVA with Dunnett’s multiple comparisons test. The p-values were determined in comparison with the data of JFH1/5AC-wt. P-values less than 0.05 were considered statistically significant.

## Supplementary information

Supplementary information
