# Supplementary material for: Impact of novel NS5A resistance-associated substitutions of hepatitis C virus detected in treatment-experienced patients
Source: Sci Rep. 2019 Apr 5;9:5722. doi: 10.1038/s41598-019-42114-z (PMC6450881; doi:10.1038/s41598-019-42114-z)
Supplement: Supplementary file 1 — Supplementary information [file 41598_2019_42114_MOESM1_ESM.pdf]

Impact of novel NS5A resistance-associated substitutions of hepatitis C virus detected in treatment-experienced patients

Sayuri Nitta[1,3], Yasuhiro Asahina*[1,2], Takanobu Kato[3], Jun Tsuchiya[1], Emi Inoue-Shinomiya[1], Ayako Sato[1], Tomoyuki Tsunoda[1], Masato Miyoshi[1], Fukiko Kawai-Kitahata[1], Miyako Murakawa[1], Yasuhiro Itsui[1], Mina Nakagawa[1], Seishin Azuma[1], Sei Kakinuma[1, 2], Hayato Hikita[4], Tetsuo Takehara[4], Mamoru Watanabe[1]

[1] Department of Gastroenterology and Hepatology, Tokyo Medical and Dental University.

[2]Department of Liver Disease Control, Tokyo Medical and Dental University.

[3]Department of Virology II, National Institute of Infectious Diseases.

[4]Department of Gastroenterology and Hepatology, Graduate School of Medicine, Osaka University

*To whom correspondence should be addressed;

Dr. Yasuhiro Asahina
Yushima 1-5-45, Bunkyo-ku, Tokyo 113-8519, Japan.
Tel: +81-3-5803-5877
Fax: +81-3-5803-0268
E-mail: asahina.gast@tmd.ac.jp

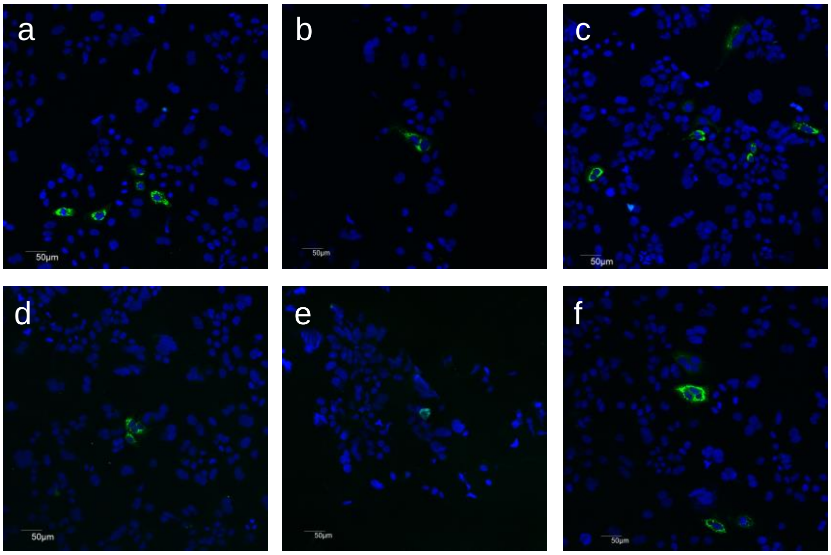

Supplementary Figure S1. Immunostaining of HCV infected cells with JFH1/5AC and its derivatives with NS5A RAS

Huh-7.5.1 cells were infected with supernatant collected from HCV-RNA transfected Huh7-25 cells. Three days after infection, indirect immunostaining was performed with an anti-core antibody. (a) - (f) indicate JFH1/5AC-wt and its derivatives as follows; JFH1/5AC (a) -wt, (b) -Q24K/L28M/R30Q/A92K, (c) -R30Q/A92K, (d) -L31F/P32del, (e) -P32del, and (f) -A92K. Scale bars indicate 50 μm.
